# In–Situ Acoustic Process Monitoring of Interfacial Events in a Moving–Tool Ultrasound–Assisted Additive Manufacturing Process

**DOI:** 10.3390/s26196234

**Published:** 2026-09-30

**Authors:** Pouria Meshki Zadeh, Shams Torabnia, Nathan Fonseca, Keng Hsu, Ehsan Dehghan-Niri

**Affiliations:** School of Manufacturing Systems and Networks, Arizona State University, 6075 Innovation Way W, Mesa, AZ 85212, USA

**Keywords:** acoustic emission, nondestructive evaluation, ultrasound-assisted additive manufacturing, solid-state joining, in-situ process monitoring, ultrasonic welding

## Abstract

Ultrasound–assisted additive manufacturing forms bonds through rapid interfacial events that are difficult to observe in situ, particularly because the deposition tool moves continuously. This study presents an energy–based acoustic framework for detecting and timing these interfacial events, demonstrated on the Resonant–Assisted Deposition (RAD) process. Rather than measuring absolute energy, the method treats the signal power of each channel as a relative indicator of acoustic energy and tracks how wave propagation is altered in structure–borne and airborne pathways, recorded by two AE sensors on the deposition head and substrate, and an airborne microphone. Because the interface acts on all pathways at once, its activity appears as a common–mode variation of the head and substrate signal powers, distinct from the opposing trade–off seen during free tool motion, and this signature locates the interfacial activities within deposition cycles. Two time–domain features segment the window: the Interfacial Slipping Duration (ISD), associated with the slip stage, and the Interfacial Consolidation Duration (ICD), the subsequent bond formation stages; together they give the interfacial–activity duration $T_{IA}$. The measured ISD is consistent with an independently reported slip stage thermal response, and the same signal–derived windows confine the time–frequency analysis to the interval where ultra–harmonic interfacial content appears. Across controlled changes in excitation frequency and substrate temperature, the features remain extractable and respond in distinguishable ways, indicating repeatability and sensitivity. The method provides a physically interpretable, fixed–sensor basis for characterizing the timing of interfacial events in a moving–tool process, intended for in–situ process monitoring.

## 1. Introduction

Ultrasonic bonding is a branch of solid–state joining processes that forms metallurgical bonds through high–frequency mechanical oscillations applied together with a compressive normal force, without inducing bulk melting. Fusion–based techniques such as Laser Powder Bed Fusion (LPBF), Directed Energy Deposition (DED), and Wire Arc Additive Manufacturing (WAAM) achieve interlayer bonding through localized melting and re–solidification, which commonly introduces solidification– and cooling–related discontinuities such as oxidation, porosity, hot cracking, residual stresses, delamination, and geometric distortion [1,2,3]. Ultrasonic processes avoid these defects by forming joints through severe plastic deformation and diffusion at the interfaces, yielding a fine microstructure with comparatively homogeneous mechanical properties [4,5]. These characteristics make ultrasonic bonding particularly advantageous for joining non–weldable or dissimilar materials, as well as for applications where micro–structural homogeneity and dimensional accuracy are critical. Processes in this family include, but are not limited to, Ultrasonic Metal Welding (USMW), ultrasonic brazing, adhesive–assisted ultrasonic bonding, and ultrasound–assisted additive manufacturing [6].

Despite these advantages, ultrasonic bonding processes are acutely sensitive to small deviations in process parameters: slight changes in vibration amplitude, normal force, excitation frequency, or surface condition can shift the interfacial regime from adequate bonding to under–welding or localized overheating, directly affecting joint strength, defect formation, and the dimensional fidelity of the resulting structure [7,8]. This sensitivity has motivated a range of in–situ monitoring approaches that aim to track the bonding process as it occurs, differing in the physical quantity they observe and in how closely that quantity reflects the state of the bonding interface itself.

The most readily available monitoring signals are those already integrated into the welding machine, including weld time, tool displacement, weld depth, and power curves, which have been widely examined for their correlation with bond quality. Several studies have investigated how material hardness, rolling direction, surface cleanliness, and overlap positioning influence these machine outputs, aiming to relate them to weld strength or defect formation [9]. It has nonetheless been consistently reported that such signals alone lack the resolution and precision required for reliable weld-state identification, particularly when distinguishing the different stages of joint formation [10].

Thermal sensing brings the measurement closer to the interface by recording the temperature evolution that accompanies welding [11]. A range of temperature–sensing devices has been employed for this purpose, including infrared systems and embedded or thin–film thermocouples [12,13,14,15]. These studies have sought to relate heat generation to joint–quality metrics such as tensile–shear load, T–peel strength, weld area, and defect formation, and have examined how weld time, clamping force, vibration amplitude, and material thermal conductivity affect the interface temperature and, in turn, bond strength and plastic deformation. The principal advantage of thermal monitoring lies in its direct relationship to frictional heating and deformation work, which makes it effective for identifying overheating, under–welding, and the onset of defects such as intermetallic formation or excessive thinning; these studies have consistently shown that optimal weld quality corresponds to a controlled temperature rise, sufficiently high to promote bonding yet low enough to avoid microstructural degradation. Thermal methods are nevertheless constrained by the spatial averaging inherent to infrared systems, the intrusiveness of embedded thermocouples, and the difficulty of placing sensors near the true interface, and, more fundamentally, they provide only an indirect, thermal proxy for the bonding event and cannot directly observe the interfacial mechanics that govern joint formation.

Mechanical waves, both structure–borne and airborne, provide a nondestructive evaluation (NDE) method that addresses these limitations. Unlike machine signals or temperature measurements, mechanical–wave sensing records the elastic waves generated by the bonding process itself. The horn, workpieces, and anvil respond to the ultrasonic excitation, and changes in bonding conditions modulate these vibrations in measurable ways [16]. Several sensing techniques have been used to capture these signatures. Laser Doppler Vibrometry measures oscillation amplitude, phase, and frequency content at the horn, terminals, or workpiece surfaces, allowing high–resolution tracking of how the vibration field evolves during welding [17,18]. Piezoelectric sensors bonded to terminals or fixtures convert local strain and mechanical vibration into electrical signals, which reflect changes in resonance conditions, internal friction, fixture compliance, and clamping [19]. These sensors respond to subtle variations in fixture stiffness, material softening, joint consolidation, and relative motion at the interface, which often appear well before quality deviations are visible in machine signals alone [8,20]. Some studies have also reported nonlinear spectral phenomena, including higher–order harmonics, modulation patterns, and sidebands around the fundamental ultrasonic frequency [21].

Beyond weld-quality prediction, a growing body of research focuses on characterizing the bond-formation process, with the aim of resolving the physical stages and governing mechanisms involved in ultrasonic joining. Several bonding mechanisms have been proposed in the literature, including mechanical interlocking, localized melting, adhesion, recrystallization, diffusion, acoustic softening, and dynamic recrystallization [22,23,24,25]. Despite differences among these models, friction and periodic shear deformation at the interface are commonly recognized as primary drivers of joint formation [26,27]. The interaction of these mechanisms can produce interfacial regimes in which the bonding surfaces undergo slipping, sticking, or transitions between the two, resulting in distinct stages of bond formation.

Optical and vision–based techniques were a means to monitor these stages. Specifically, high–speed imaging has been used to directly observe the evolution of the weld interface and identify transitions between successive bonding stages, providing visual evidence of mechanisms otherwise inferred from indirect measurements [28,29]. Such techniques offer high spatial and temporal resolution and provide a direct means of examining interfacial behavior, but their applicability depends on maintaining optical access to the region of interest. Laser Doppler vibrometry has also been employed to measure vibration amplitude and relative motion among the horn, workpieces, and anvil, enabling the slip and stick behavior at the interface to be characterized from their relative mechanical response [30].

These studies, however, were mainly conducted on conventional USMW setups, in which the horn and workpieces remain stationary relative to the sensing equipment. Such configurations are well suited to stationary joining processes but do not transfer directly to ultrasound–assisted additive manufacturing, where the deposition tool moves continuously across the build area. The tool motion limits thermal and point–wise vibration measurements that rely on maintaining a fixed measurement location relative to the region of interest. Optical techniques face a different constraint. Although they are likely to provide higher spatial resolution among these modalities and observe the interface most directly, characterizing bond formation in an additive process would require the imaging system to follow the moving tool while maintaining a suitable side view of the deposition region. This becomes increasingly difficult as the build progresses, since the growing structure can progressively obstruct the line of sight, particularly for geometrically complex parts. A suitable monitoring approach must therefore accommodate the moving tool, sense an interface buried beneath it, remain applicable as the part geometry develops, and still resolve the mechanically driven interfacial activities associated with bond formation. Acoustic sensing can satisfy these requirements, since fixed sensors mounted on the machine structure and substrate record the propagating mechanical waves without following the tool. How these signals should be analyzed to isolate and characterize interfacial events in a moving–tool process nevertheless remains an open question.

To address this, the present study develops an energy–based acoustic framework for detecting and timing interfacial activity during ultrasound–assisted additive manufacturing, demonstrated on the Resonant–Assisted Deposition (RAD) process. Acoustic emission sensors fixed to the deposition head and substrate, together with an airborne microphone, record the waves radiated as the tool deposits wire feedstock onto the substrate. From the signal power of each channel, the method locates the interval in every deposition cycle during which the interface is acoustically active, identified from the correlated, common–mode variation of the head and substrate powers, and extracts two time–domain features that mark its successive stages: the Interfacial Slipping Duration (ISD) and the Interfacial Consolidation Duration (ICD). The same intervals confine the time–frequency analysis to this window, where ultra–harmonic content is shown to flag interfacial activity. The features are evaluated for repeatability across repeated deposits and for their response to controlled changes in excitation frequency and substrate temperature. The aim is to establish a set of physically interpretable acoustic features that detect and time interfacial events in a moving–tool process, providing a basis for in–situ acoustic process monitoring of ultrasound–based manufacturing.

## 2. RAD Process and Machine

**Figure 1:**
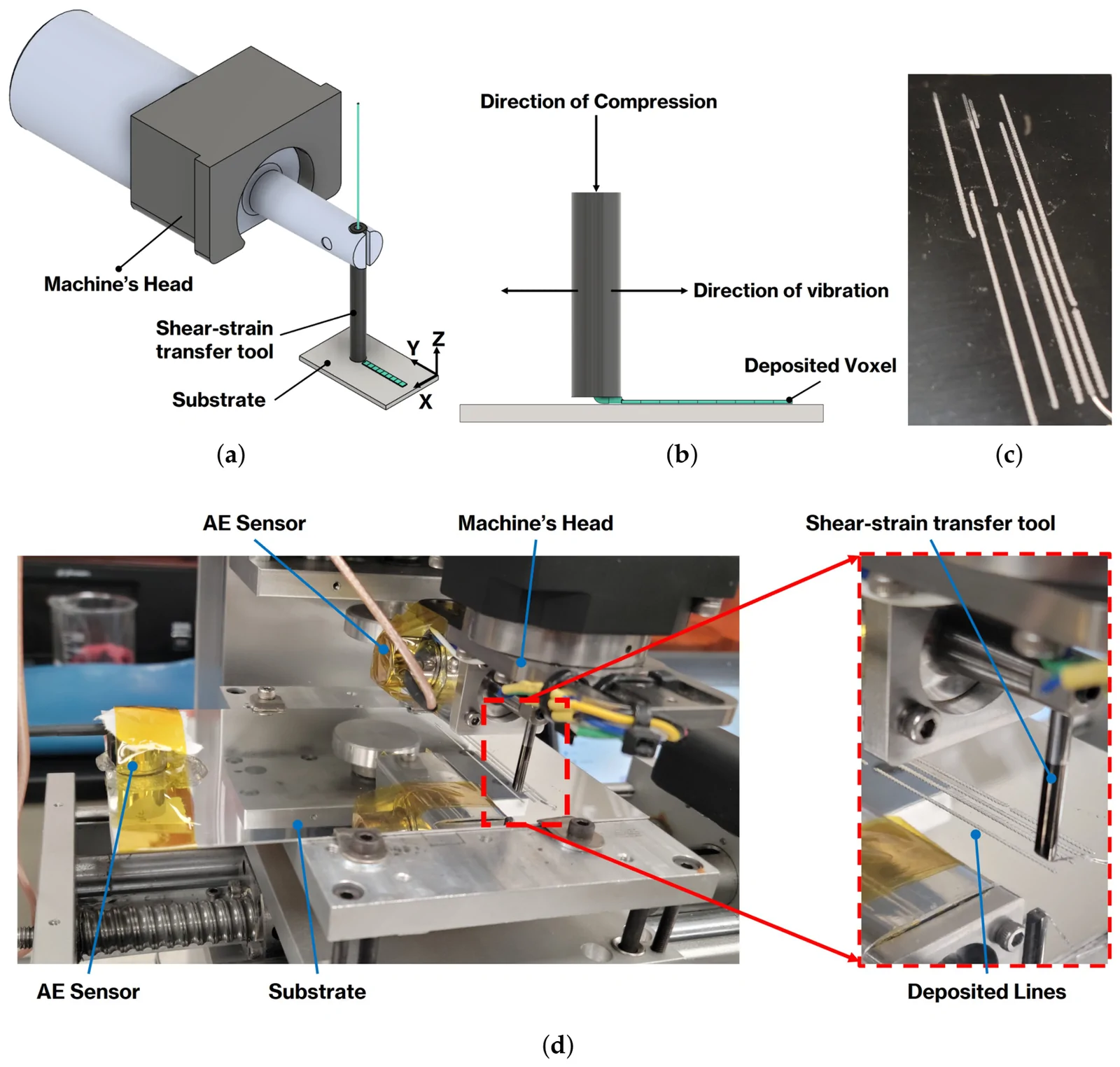
The RAD process and the deposition setup. (**a**) The deposition head delivers high–frequency mechanical vibrations through a piezoelectric converter while steering the fine wire feedstock toward the substrate. (**b**) A close–up view of the tool–substrate interaction during a deposition cycle, showing the directions of compression and vibration and the voxel formed beneath the tool. (**c**) Photograph of deposited lines on an aluminum substrate, each formed by a series of overlapping voxels. (**d**) Photograph of the RAD machine, showing the deposition head, the machine components, the two AE sensors, and deposited lines. The dashed box marks the region enlarged on the right, in which the tool tip, the substrate, and the deposited lines are visible.

RAD is an ultrasound–assisted solid–state additive manufacturing process that plastically shapes and joins metal feedstock without melting [31]. In RAD, a fine metal wire is incrementally deformed into small deposit units (voxels) and bonded to a preheated substrate or prior layers through high–frequency mechanical vibrations. In the present study, 1199 aluminum wire with a diameter of 0.35 mm is deposited onto a 15 × 30 cm 6061-T6 aluminum substrate. Unlike extrusion–based processes such as fused deposition modeling (FDM), in which a thermoplastic filament is heated and deposited as a continuous softened or molten bead, RAD keeps the metal wire in the solid state and forms discrete voxels through plastic deformation. The preheating alleviates residual stresses through mild on–the–fly annealing. The vibrations supplied by a piezoelectric ultrasonic converter induce acoustoplasticity, an effect in which oscillatory shear strains reduce the material’s flow stress and enhance interfacial diffusion, enabling metallurgical bonding with minimal heat input [32,33]. This solid–state bonding brings the advantages of ultrasonic joining discussed in Section 1, including the joining of non–weldable materials, microstructural homogeneity, and dimensional accuracy, to the layer–by–layer fabrication of metal parts. Three–dimensional geometries are created by coordinated motion of the build plate along the X–Y axes, while the Z–axis tool motion is synchronized with the programmed toolpath. The overall RAD setup and the tool–substrate interaction during a deposition cycle are illustrated in Figure 1.

Each voxel is produced in a deposition cycle that repeats along the programmed sawtooth toolpath at a nominal rate of 3 Hz. The cycle consists of three steps: (1) the tool moves to the next deposition location at a safe height, with the wire aligned slightly above the substrate or the previous layer; (2) the tool descends and presses the wire against the substrate, where the combined compressive load and oscillatory shear transform the wire into a deposited voxel and form a solid–state bond, as shown in Figure 1b; and (3) the tool retracts from the deposited voxel while moving to the following location. The ultrasonic converter drives the tool throughout the cycle, so the tool vibrates freely during the first and third steps and is in contact with the feedstock and substrate only during the second step.

Joint formation in RAD follows stages similar to those observed in USMW. It begins when the vibrating horn, typically operating within the 30–60 kHz range, shears away surface contaminants and oxides by slipping the feedstock over the surface of the workpiece (substrate), generating localized frictional heating [34]. As the cleaned surfaces achieve sufficient intimate contact and the oscillatory shear reduces the local yield strength, the material adheres to the substrate, thereby initiating the nucleation of a solid–state weld [35]. The transition between slip and stick may include an intermediate period in which both occur simultaneously [22]. When the ultrasonic vibration and compressive load are removed, the materials undergo rapid elastic recovery. Because all participating components are under compressive strain during bonding, removing the tool releases stored elastic energy, producing a slight spring–back at the interface.

The controllable machine parameters in this process include the ultrasonic converter’s center frequency and the substrate temperature. Variations in excitation frequency modify the dynamic response of the tool–workpiece system and can influence how acoustic energy couples into the interface during the slipping and consolidation stages. Similarly, changes in substrate temperature affect material flow stress, interfacial friction, and the extent of plastic deformation. Therefore, any alteration to these parameters can impact the progression of slip, stick, elastic recovery, and joint formation.

## 3. Acoustic Sensing Framework

### 3.1. Acoustic Sources and Propagation Pathways

The principles of RAD described in Section 2 make clear that any monitoring technique requiring a fixed field of view or continuous focus on a single component is ill–suited to this process, because the deposition head moves continuously and the bond forms at a buried, moving interface. Processes that use high–frequency ultrasonic excitation to join components inherently emit stress waves across distinct frequency bands, governed by the resonance of the ultrasonic converter and by the localized tool–feedstock–substrate interactions. A monitoring approach that senses these waves as they propagate, rather than one that images a fixed region, is therefore better matched to the process.

The ultrasonic converter, as the first source of acoustic generation in RAD process, drives the deposition tool at high frequency and produces a continuous acoustic field associated with the driven vibration of the system. The second source is mechanical interactions at the tool–feedstock–substrate interface, which creates a transient acoustic field as the interface evolves through slipping, the transition from slipping to sticking, plastic deformation, and elastic recovery during each cycle. These mechanical interactions are likely to leave a twofold effect on the acoustic field: generation of new mechanical waves, and altering the converter–driven acoustic field by changing the mechanical boundary conditions at the contact. Because this contribution exists only while the tool, feedstock, and substrate interact, the onset and evolution of interfacial activities are expected to produce temporary changes in the measured acoustic response.

**Figure 2:**
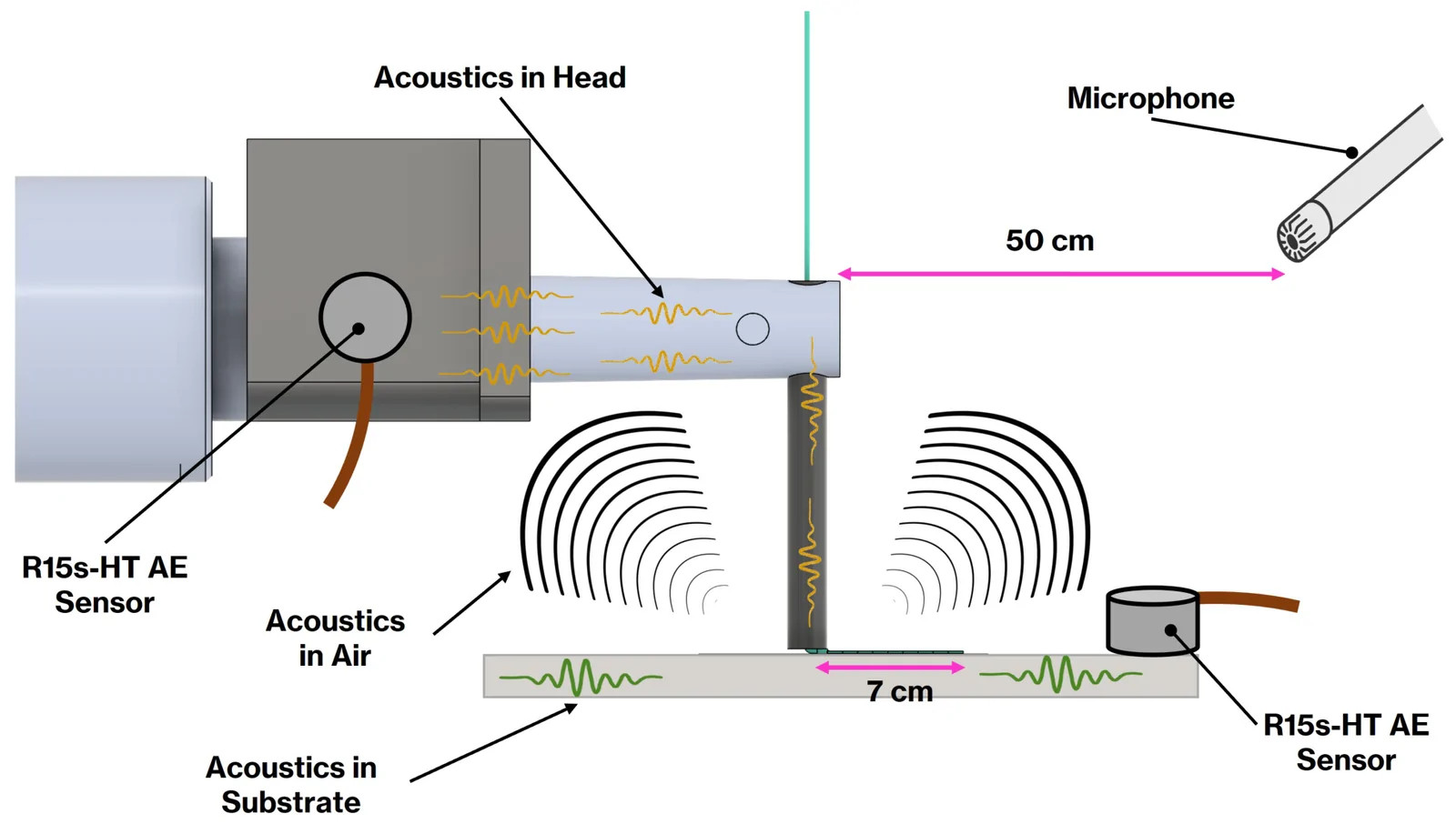
Sensing framework and expected acoustic propagation paths in the RAD process. Structure–borne acoustic waves generated by the ultrasonic converter travel through the deposition head and into the substrate, where they are captured by AE sensors mounted on the head and substrate. At the same time, airborne acoustic waves radiate into the surrounding space and are recorded by a microphone positioned near the tool.

Once generated, these waves propagate through multiple media. Structure–borne waves travel through the deposition head and, when mechanical coupling is established, across the interface into the substrate, while vibration of the system radiates into the surrounding air. These pathways are illustrated in Figure 2, and the sensing configuration mirrors them: an AE sensor on the deposition head, an AE sensor on the substrate, and an airborne microphone.

### 3.2. Signal Power as a Relative Indicator

The response measured along each pathway reflects both contributions, so a change in the mechanical state of the interface can leave its mark on the measured signals in several ways: on their amplitude and energy, on their frequency content, and on how these quantities are distributed between the sensing pathways over time. While the spectral variations is extracted form the recorded signals, the acoustic energy carried by each pathway cannot be measured directly. Each structural component, mounting boundary, coupling condition, and acquisition chain acts as a transfer function on the waveform, and attenuation and source-to-sensor distance change as the head moves. For this reason, the framework does not attempt to recover energy in joules or to close a calibrated physical energy balance.

What the measurement does preserve is the relationship between the energy carried along a pathway and the energy of the recorded signal. As long as the propagation and measurement conditions of a given channel remain fixed, a change in the acoustic energy reaching the sensor produces a proportional change in the signal energy, so the latter tracks the former up to an unknown scaling factor. Signal energy is therefore used here as a relative, uncalibrated indicator of the acoustic energy on each pathway. For the *i*-th sensing channel, the measured signal energy over a time interval $\left[t_{0},t_{1}\right]$ is defined as

(1) $$E_{i}=\int_{t_{0}}^{t_{1}}V_{i}^{2}\left(t\right)\;dt,$$

where $V_{i}\left(t\right)$ is the recorded voltage waveform. Accordingly, $E_{i}$ is used to characterize relative changes in the measured acoustic response, and its value is not compared across channels as an absolute quantity.

Since the interfacial activity occupies only a short part of each deposition cycle, the quantity of interest is not the energy accumulated over an interval but its evolution in time. Removing the integral from Equation (1) leaves the instantaneous voltage–based signal power,

(2) $$P_{i}\left(t\right)=V_{i}^{2}\left(t\right),$$

which is simply the square of the signal amplitude and therefore available at every sample of the record. Its integral over any interval returns the signal energy of that interval, so $P_{i}\left(t\right)$ carries the same relative information as $E_{i}$ while resolving when that energy arrives.

The squared waveform, however, still oscillates at twice the excitation frequency, whereas the interfacial activity evolves over milliseconds. To retain the slower variations and suppress the carrier, a moving average is applied to the squared waveform. In discrete form,

(3) $${\tilde{P}}_{i}\left[n\right]=\frac{1}{w}\sum_{k=n-w/2}^{n+w/2}P_{i}\left[k\right],$$

where *w* is the averaging–window length. In the present analysis, *w* = 10,000 samples, which corresponds to 5 ms at the 2 MS/s sampling rate used in this study (Section 4). This window is short relative to the deposition cycle of approximately 360 ms, so the cycle–scale variations in signal power associated with changes in the mechanical state of the RAD system are retained. The resulting ${\tilde{P}}_{i}\left[n\right]$ profiles can therefore be used to evaluate the relative energy evolution measured along each sensing pathway.

### 3.3. Segmentation of the Deposition Cycle

Comparing these profiles across pathways, rather than reading any one in isolation, is central to the framework, because a variation at a single sensor can equally arise from propagation distance, attenuation, or local coupling. The two contributions, however, are expected to act on the pathways differently. The converter–driven field is redistributive: as contact is made and broken, acoustic propagation intensity is exchanged between the head and substrate, and the two structure–borne channels tend to vary in opposition. Interfacial activity, by contrast, acts on both pathways at once, dissipating or radiating energy at the contact, so its signature is expected to be a common variation of the head and substrate responses rather than a trade–off between them. The segmentation criterion below uses this distinction to locate the interfacial activity against the persistent converter background, and the experimental evidence supporting it is discussed in Section 6.

The segmentation is applied to each deposition cycle separately. A cycle is isolated between two consecutive maxima of the head power, which recur at the deposition period, and the smoothed powers of the head and substrate channels, ${\tilde{P}}_{h}\left[n\right]$ and ${\tilde{P}}_{s}\left[n\right]$, are normalized within the cycle. The microphone channel is not used for segmentation, which is discussed with details in Section 5.

Considering the variation of computed signal powers, each cycle is divided into two general phases windows: Retained and Coupling, which are first separated using the normalized difference of the two powers,

(4) $$\Delta \left[n\right]=\frac{{\tilde{P}}_{h}\left[n\right]-{\tilde{P}}_{s}\left[n\right]}{{\tilde{P}}_{h}\left[n\right]+{\tilde{P}}_{s}\left[n\right]},$$

where *n* is the sample number within the cycle. The Retained window, in which the head carries the larger share of the measured power, corresponds to Δ[n]>0, and the Coupling window, in which the substrate carries the larger share, corresponds to Δ[n]<0. Because Δ[n] is bounded between −1 and 1, it describes how the measured power is partitioned between the two pathways, independent of its overall level.

For the same reason, the difference cannot reveal the interfacial contribution, since a proportional rise or fall in both powers leaves it unchanged. Within the Coupling window, the transitions are therefore located on the total power,

(5) $${\tilde{P}}_{t}\left[n\right]={\tilde{P}}_{h}\left[n\right]+{\tilde{P}}_{s}\left[n\right],$$

which reflects how much power reaches the two pathways rather than how it is shared between them. Three points are defined on ${\tilde{P}}_{t}$: point *D* is its absolute maximum within the Coupling window, point *B* is the first local maximum preceding *D* with ${\tilde{P}}_{t}\left[B\right]/{\tilde{P}}_{t}\left[D\right]>0.5$, and point *C* is the minimum between *B* and *D*. The ratio condition ensures that low–amplitude local maxima in the total power are not selected as *B*. The extracted points set the boundary of two new features:

(6) $$\mathrm{ISD}=t_{C}-t_{B},\;\mathrm{ICD}=t_{D}-t_{C},\;T_{IA}=\mathrm{ISD}+\mathrm{ICD}.$$

The *B*–*C* interval is termed the Interfacial Slipping Duration (ISD), and the *C*–*D* interval is named as the Interfacial Consolidation Duration (ICD); together they give the interfacial–activity duration $T_{IA}$. These names refer to the interfacial stages described in Section 2 with which the two intervals are associated, and the basis for this association is presented in Section 6. Also, the characteristics of the power variation within these windows are thoroughly investigated in Section 5.

Since all three points are located by fixed rules on a single derived curve, the proposed power–derived features, ISD and ICD, are obtained without operator intervention, making them suitable for online process monitoring application. The position of these points on the averaged baseline cycle is shown in Section 5.1, and the extraction success rate for each process condition is reported in Section 5.3.

## 4. Experimental Setup

In this contribution, the RAD machine was used to bond 1199 aluminum wire to a 6061–T6 aluminum substrate under three process conditions: Baseline Condition (BC), Frequency–Shifted condition (FS), and Temperature–Shifted condition (TS). The BC includes setting the converter center frequency at 54.5 kHz and preheating the build plate up to approximately 100 °C. In the FS condition, the converter is forced to excite at 54 kHz, while in the TS condition, the substrate temperature was lowered to 70 °C. All other process parameters were held constant. Each condition was repeated five times, with five individual lines, each approximately 7 mm in length, printed per condition, to assess repeatability and consistency of the observed acoustic and proposed features. Printing each line was performed at a deposition rate of 3 Hz, which defines the synchronized Z–axis tool motion and planar movement of the substrate, and lasted about 4.2 s, consisting of multiple deposition cycles.

Acoustic data were captured for all five attempts through airborne and structure–borne pathways using two independent acquisition systems. The two systems were not synchronized in hardware; data collection began before each print attempt and continued beyond the print duration. Synchronization was later performed in MATLAB 2026a by aligning the signals at their mutual start point. The initial 0.7 s of each recording, corresponding to converter activation and stabilization, was removed prior to analysis.

Airborne acoustic waves were recorded using a PCB 378C01 microphone, a PCB 480B21 signal conditioner (PCB Piezotronics, Inc., Depew, NY, USA), and an NI–9775 digitizer integrated into an NI–9173 c–DAQ chassis (National Instruments Corporation, Austin, TX, USA). The 378C01 microphone provides a flat frequency response from 4 to 100 kHz with a sensitivity variation of ±3 dB. A sampling rate of 2 MS/s was selected. The microphone was positioned approximately 50 cm from the tool at an angle of 330°, following the placement recommendations reported by Larimer [36]. Both the distance and angle were defined with respect to the tool’s position at the center of the print area and naturally vary as the tool moves during deposition. The sensor placement strategy is illustrated in the framework section.

Structure–borne acoustic signals were recorded using a MISTRAS Micro–II Express chassis (MISTRAS Group, Inc., Princeton Junction, NJ, USA) equipped with two MISTRAS R15s–HT sensors. These sensors were mounted on the machine head and substrate using tape to ensure stable coupling during printing. The analog signals were amplified using a 2/4/6 MISTRAS voltage preamplifier set to 20 dB gain, and waveform streaming was configured with a zero–second buffer to ensure uninterrupted acquisition. A sampling rate of 2 MS/s was used, matching that of the airborne system. Because the deposition occurs near 100 °C, a high–temperature ultrasonic couplant (HiTempco+; Echo Ultrasonics, Inc., Bellingham, WA, USA), rated up to 455 °C, was applied to maintain reliable acoustic transmission between the sensors and the machine surfaces. The couplant was renewed and both sensors were re–taped before each process condition, and their condition was inspected after each print attempt.

## 5. Results

The results section first focuses on the BC scenario to establish the acoustic signature of the deposition cycle and to extract the developed features defined in Section 3.3. The FS and TS conditions are subsequently used to assess the repeatability of these features and their sensitivity to changes in process parameters. For each condition, five print attempts were performed, and consistent trends in signal power and segmentation were observed across all trials. Therefore, for clarity, the results presented for each condition correspond to a single representative print attempt, while the repeatability of the extracted features across the five attempts is reported in Section 5.3. The physical interpretation of these observations is discussed in Section 6.

**Figure 3:**
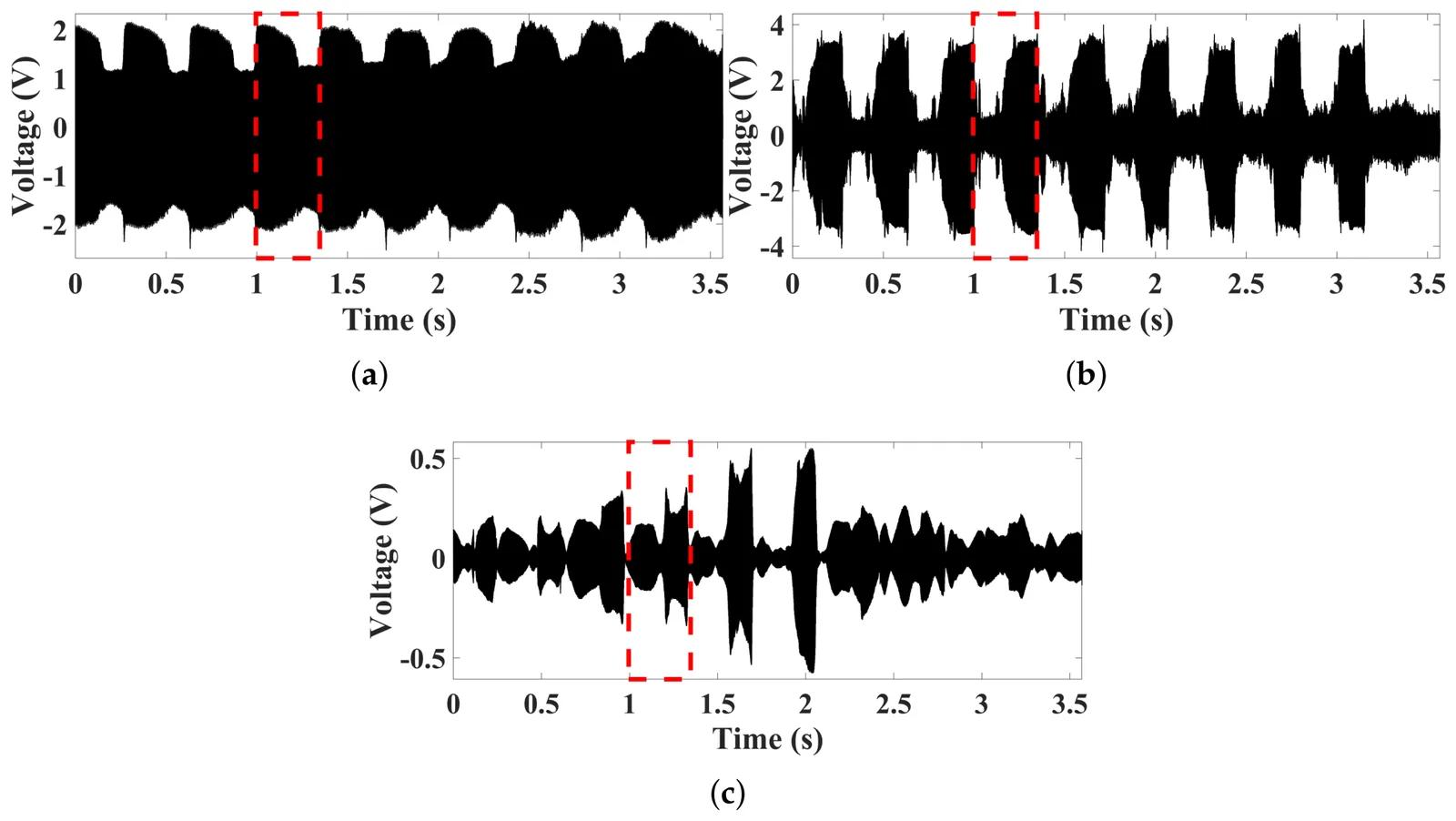
Time–domain acoustic signals recorded by (**a**) the head–mounted AE sensor, (**b**) the substrate–mounted AE sensor, and (**c**) the microphone during the RAD process. The red dashed box identifies a representative complete deposition cycle, spanning one Retained window and one Coupling window.

The captured waveforms provide the most direct view of the cyclic tool–substrate interactions in RAD and form the basis for the energy–based and frequency–domain analyses that follow. Figure 3 shows the entire collected signals, in which a clear correlation between the AE channels can be observed: the drop in head AE amplitude is accompanied by a rise in substrate AE amplitude. Each deposition cycle can therefore be viewed as a window during which the head vibrates freely, with a high head AE amplitude, followed by a window in which the vibration is disturbed by contact with the substrate and the substrate AE amplitude is high. These correspond to the Retained and Coupling windows defined in Section 3.3, which are extracted from the signal powers of the two channels in Section 5.1. A segment spanning one of each, marked by the dashed box in Figure 3, captures a full cycle. The sharp peaks in the head signal occur at intervals of about 0.36 s, in good agreement with the theoretical deposition rate of 3 Hz.

The microphone signal also exhibits temporal correlation with the structure–borne acoustic data. In the print attempt presented here, the peaks in the microphone data generally coincide with the Coupling windows within the first two seconds of the process. Within this period, the microphone waveforms become stronger as the head moves closer to the sensor, reaching the minimum distance at approximately 2 s. In contrast, during the final 1.5 s, the airborne acoustic response reflects a mixture of head and substrate AE activities, accompanied by a gradual reduction in amplitude. Upon reviewing the recorded video, the overall decrease in amplitude can be attributed to the head moving farther from the microphone, thereby increasing the propagation distance. These findings confirm that the microphone response is highly dependent on its spatial relationship with the tool, suggesting that more accurate time–resolved interpretation may require improved microphone positioning.

### 5.1. Evaluation of Signal Powers and Feature Extraction

**Figure 4:**
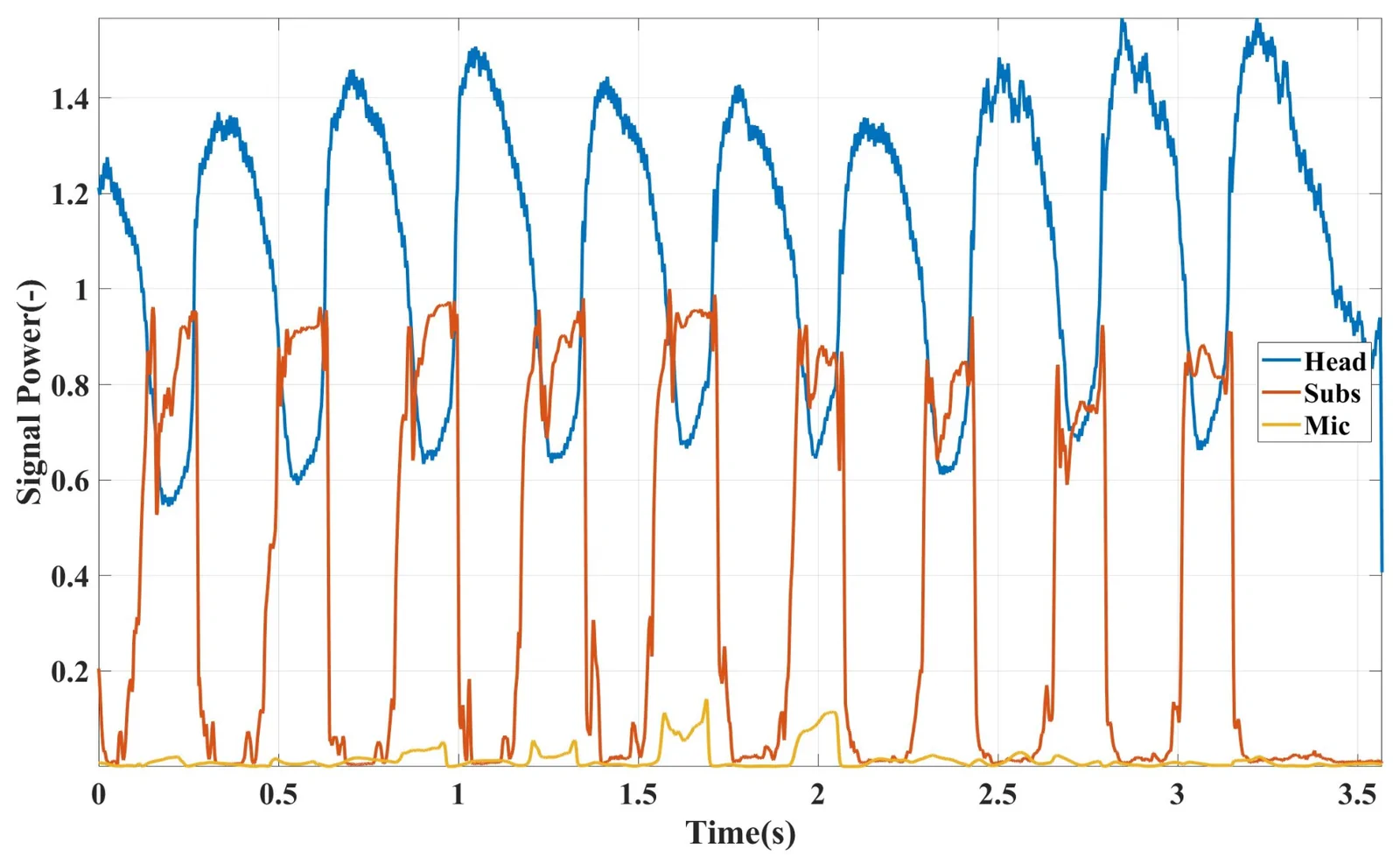
Normalized signal power recorded by head–mounted (blue), substrate–mounted (orange), and airborne (yellow) sensors during the RAD process. The alternating peaks correspond to the Retained and Coupling phases of successive deposition cycles.

The signal waveforms provide a general view of the process, but their amplitudes fluctuate for many reasons and are difficult to read directly. The smoothed signal power suppresses the high–frequency oscillations and emphasizes the timing and redistribution of energy between the head and substrate within each cycle. As shown in Figure 4, this reveals a repeatable pattern that can be used to track the stages of interfacial activity.

The Retained and Coupling phases identified in the waveforms are resolved far more clearly in the power curves, where they correspond to positive and negative values of Δ[n] in Equation (4), respectively. The head power curve never approaches zero throughout the process, and its peak value varies among the Retained windows.

Regarding the microphone power curve, between approximately 0.8 and 2.2 s the microphone signal exhibits temporal correlation with the substrate power curve, while remaining relatively flat elsewhere. This is consistent with the spatial dependence observed in the waveforms, and the airborne data were therefore excluded from the segmentation.

**Figure 5:**
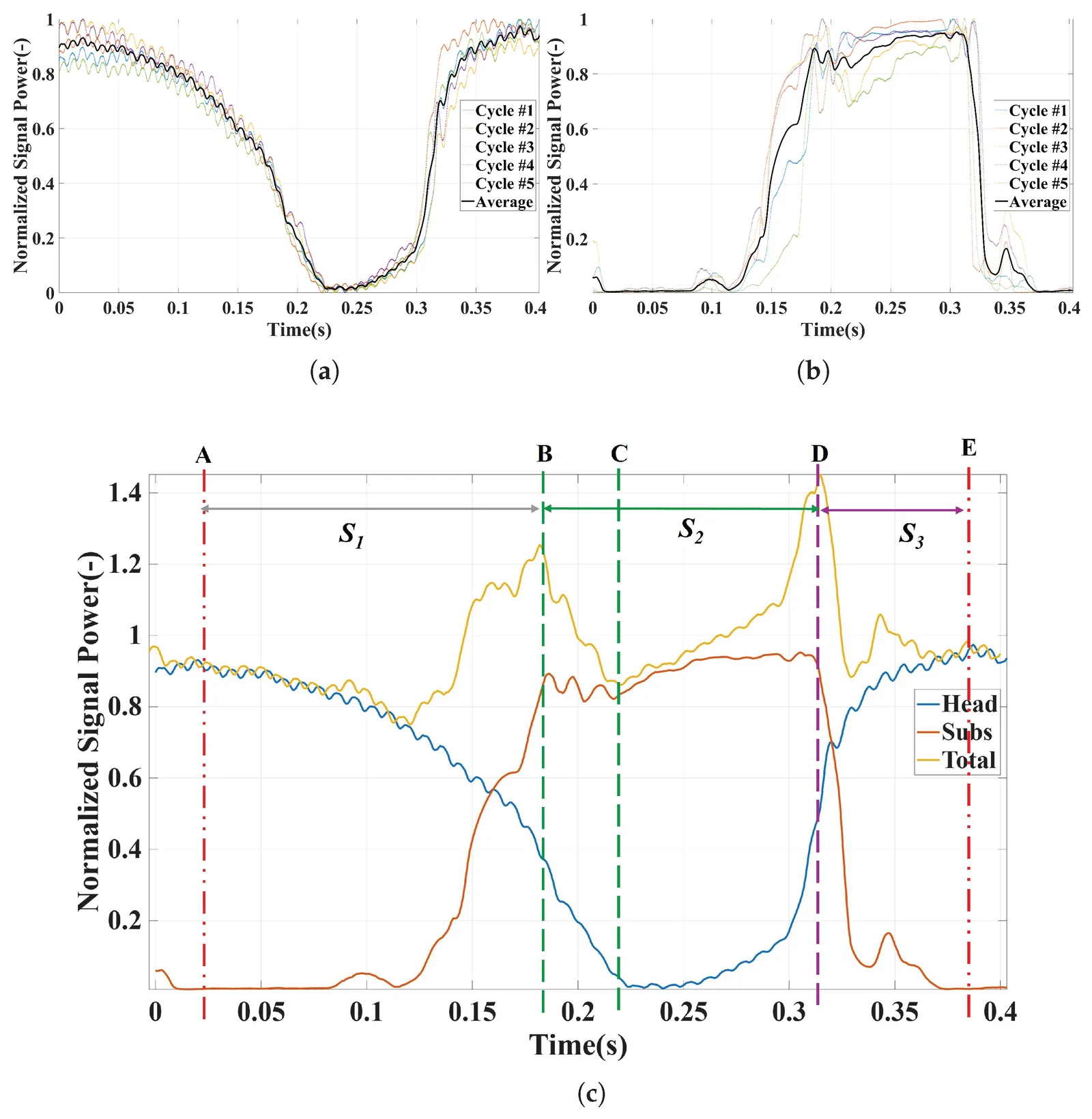
The power curves of five deposition cycles acquired from structure–borne signals. (**a**) The power curves of AE data from the sensor attached to the machine’s head, (**b**) from the sensor attached to the substrate, and (**c**) the average of the power curves.

To characterize the typical evolution of the signal power across deposition cycles, the power curves of five cycles were isolated between consecutive maxima of the head power, as described in Section 3.3. The head power was additionally offset–corrected to remove its constant level, so that the transient power variations within the cycle could be compared directly. The five normalized profiles of each channel are presented in Figure 5a,b, and their averages are shown in Figure 5c, together with the total power ${\tilde{P}}_{t}$ in yellow. It should be noted that the average interval of the isolated cycles was approximately 0.36 s, consistent with the theoretical deposition frequency of 3 Hz.

Figure 5c can be divided into three general zones, denoted $S_{1}$, $S_{2}$, and $S_{3}$. Points *A* and *E* refer to the beginning and end of the averaged cycle, and points *B*, *C*, and *D* are located on ${\tilde{P}}_{t}$ using the criterion described in Section 3.3. Outside the *B*–*D* interval, the head and substrate powers follow the characteristic alternating trend, in which a rise in one is accompanied by a drop in the other.

Within $S_{1}$, the head power decreases with varying slopes, whereas the substrate power increases. Early in $S_{1}$, the head power continuously decreases while the substrate curve stays low and steady. Later the drop in head power coincide with rise in substrate power, marking the transition from the Retained window into the Coupling window.

Zone $S_{2}$ spans *B* to *D*, where this trade–off disappears. Between *B* and *C*, both curves decrease together, the head sharply and the substrate with mild downward fluctuations, so that the total power falls to its minimum at *C*. This interval lasts about 32 ms and coincides with the portion of the programmed toolpath in which the tool is commanded into contact with the substrate. From *C* to *D*, both curves reverse and rise together, and the total power reaches its maximum within the Coupling window at *D*. This interval spans about 96 ms.

In $S_{3}$, after *D*, the head and substrate powers resume their opposing trend, which continues until the end of the cycle.

For the averaged baseline cycle, the segmentation therefore gives an ISD of about 32 ms and an ICD of about 96 ms, corresponding to a common decrease followed by a common increase in the head and substrate powers within the Coupling window. These values come from the averaged cycles in Figure 5c and serve only to illustrate the segmentation; the ISD and ICD for all three conditions are quantified across every cycle and print attempt in Section 5.3.

### 5.2. Spectral and Time–Frequency Analysis

This section examines the spectral content of the acoustic signals to determine whether the windows extracted from the signal power coincide with changes in spectral content, and to distinguish the changes observed during tool–substrate interaction from those observed during free tool motion.

**Figure 6:**
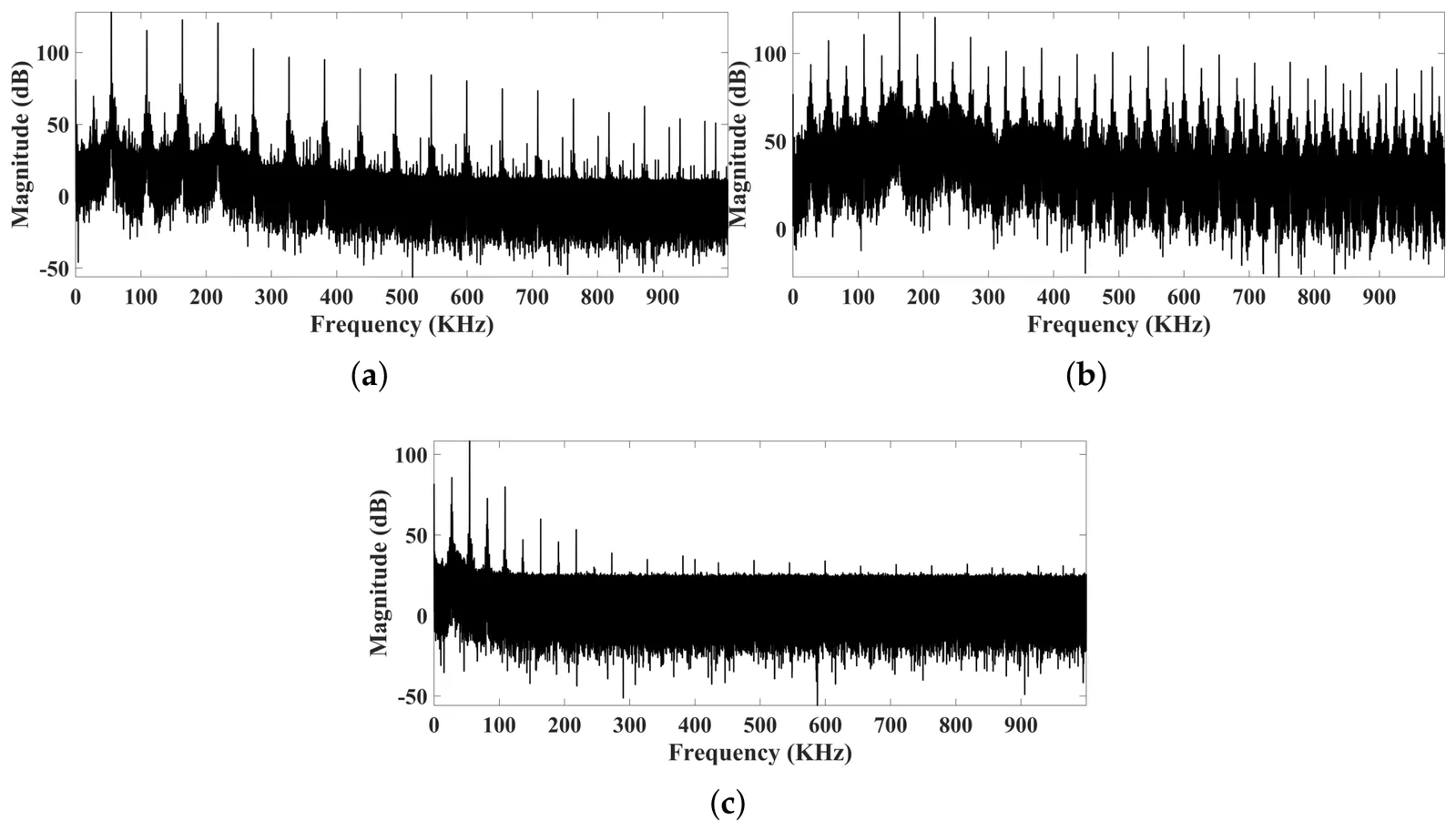
Spectral response of acoustic signals acquired from (**a**) the head–mounted AE sensor, (**b**) the substrate–mounted AE sensor, and (**c**) the microphone.

The spectral analysis of the structure–borne and airborne acoustic data, presented in Figure 6, shows the converter’s fundamental frequency (54.5 kHz) and its harmonic structure under the baseline condition. Additional peaks are also observed that do not correspond to integer multiples of the excitation frequency. These ultra–harmonics can be described generally as $f_{uh}=f_{sub}+nf_{0}$, where $f_{sub}$ is the sub–harmonic, $f_{0}$ is the fundamental frequency, and *n* is an integer. Such ultra–harmonics are observed at several frequency bins, including 27.25, 81.75, and 136.25 kHz, across all FFT plots.

**Figure 7:**
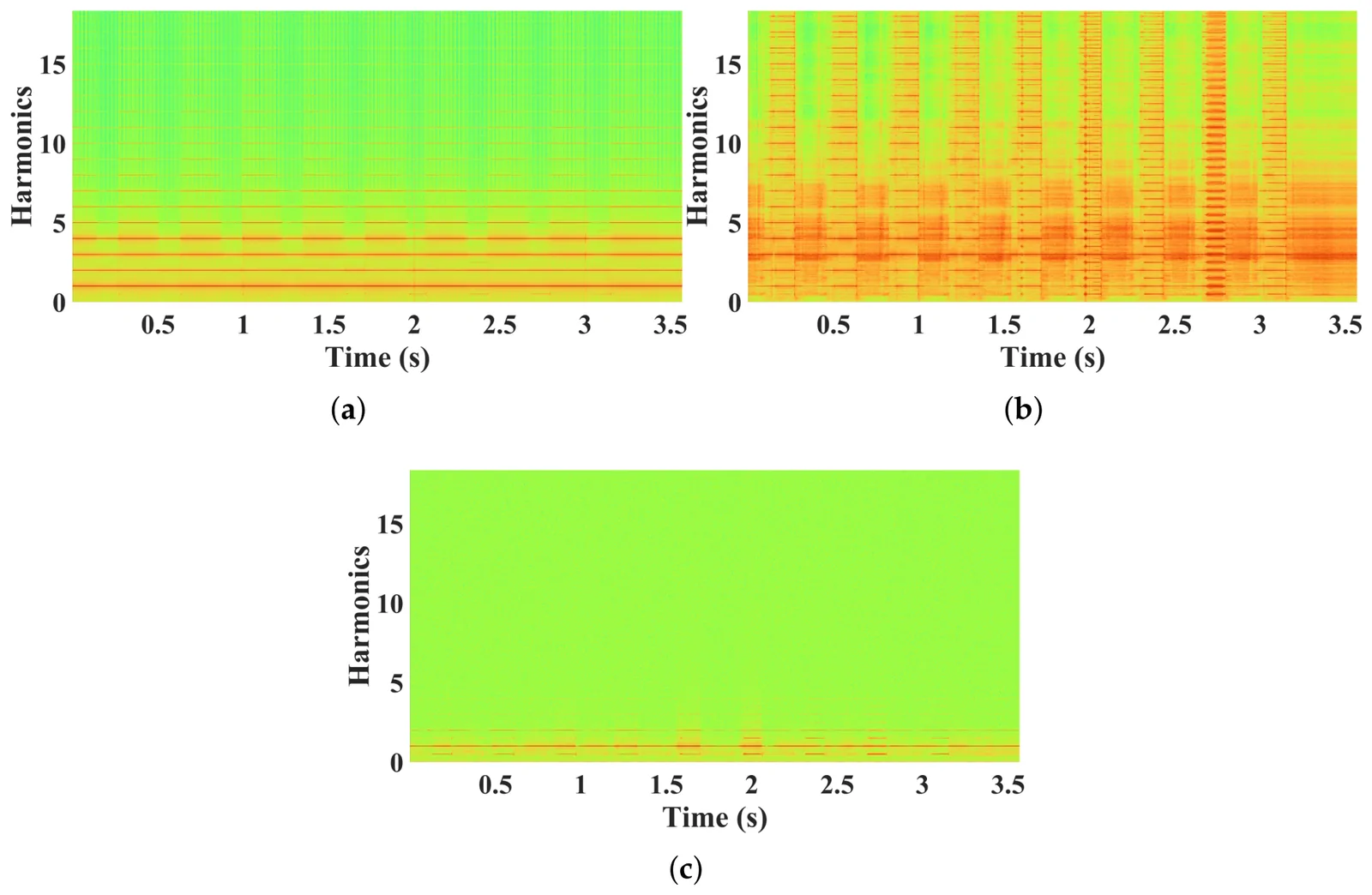
Time–frequency representation of acoustic signals obtained via Short–Time Fourier Transform (STFT) for (**a**) the head–mounted AE sensor, (**b**) the substrate–mounted AE sensor, and (**c**) the microphone. The harmonic distribution and intensity evolution show distinct spectral behaviors during Retained and Coupling phases.

Understanding the overall spectral pattern is essential; however, the evolution of frequency components over time can only be resolved through time–frequency analysis. To achieve this, the recorded signals were processed using a Short–Time Fourier Transform (STFT), and the resulting spectrograms are shown in Figure 7. The vertical axis represents the number of harmonics, derived by normalizing frequency bins by the converter’s excitation frequency of 54.5 kHz.

The spectrograms combine the time– and frequency–domain behavior in a single view, and the cyclic structure of the process is clearly visible in all channels. Harmonics is the common feature between the two identified phases within each deposition cycle. During the Retained phases, the head spectrogram shows strong and stable harmonic bands while these components are weaker in the substrate response; during the Coupling phases, this distribution inverts, with the substrate becoming richer in harmonics as the head intensity falls. This inversion follows the alternating trend of the power curves in Section 5.1. Ultra–harmonics, in contrast, appear only within the Coupling phases and are most pronounced in the substrate channel. These frequency bins also vary between nominally identical cycles. Across the all Coupling windows of a single BC print, the ultra–harmonic peaks in the substrate signal fluctuate considerably, whereas the integer harmonics remain almost constant. Additional spectral patterns beyond the harmonics and ultra–harmonics also appear during the Coupling windows, recurring from cycle to cycle without being identical.

The microphone spectrogram in Figure 7c also shows process–related content. Around 1.6 and 2 s, four distinct frequency bins can be identified: the first harmonic (fundamental frequency), the first ultra–harmonic (27.25 kHz), the second harmonic (109 kHz), and the second ultra–harmonic (81.75 kHz). The same pattern repeats multiple times within the spectrogram, consistent with the spatial dependence of the airborne response noted earlier.

**Figure 8:**
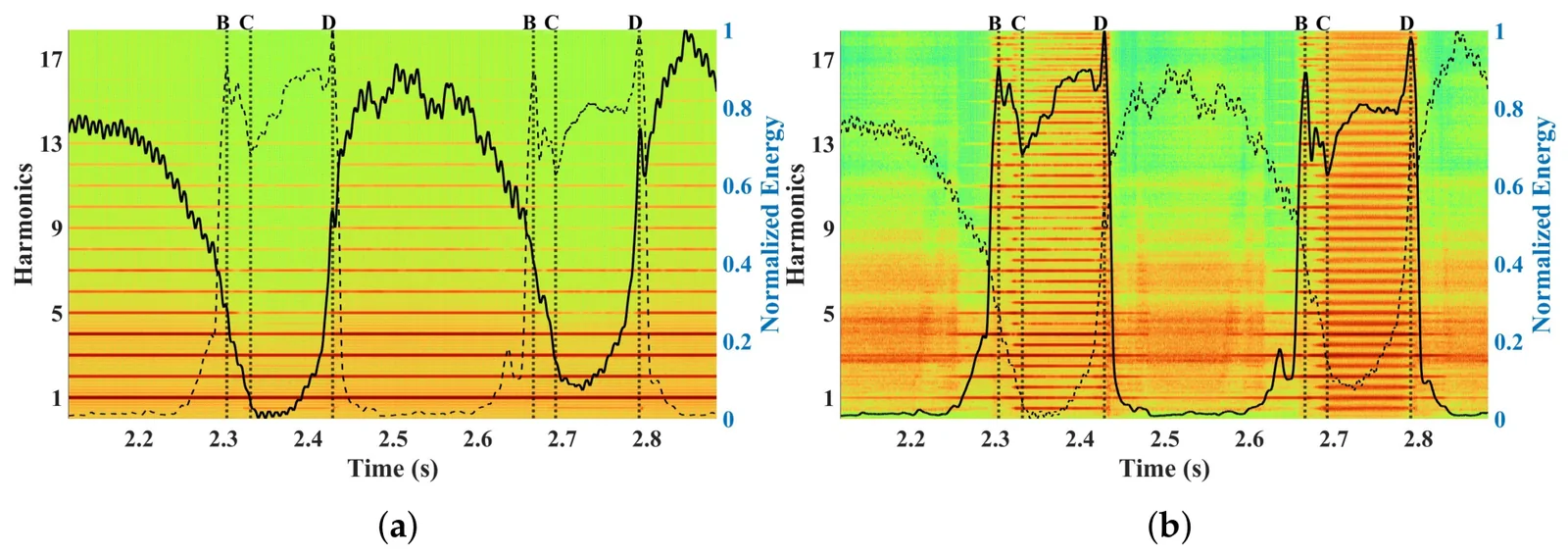
Cycle–specific spectrograms extracted from (**a**) head–mounted and (**b**) substrate–mounted AE signals with overlaid normalized power profiles, with the ISD and ICD intervals marked by points *B*, *C*, and *D*.

The full spectrograms cover the entire deposition cycle, including the intervals of tool motion outside the Coupling window. To examine the spectral content within the interfacial–activity window, the ISD and ICD boundaries extracted from the signal power were applied to the spectrograms. Figure 8 overlays the normalized power curves on the spectrograms of two consecutive deposition cycles, with the intervals marked using the same *B*, *C*, and *D* notation as in Figure 5c.

During the *B*–*C* interval corresponding to the ISD, the number of harmonics in the head signal decreases while it increases in the substrate. Toward the end of this interval, ultra–harmonics appear in both the head and substrate spectrograms. In the subsequent *C*–*D* interval (ICD), the ultra–harmonics persist, especially in the substrate spectrograms, and the harmonic distribution is more stable than during the ISD, with only minor variations within the window.

### 5.3. Feature Repeatability and Sensitivity Under Process Variations

For the ISD and ICD to be useful as monitoring features, two properties must hold: they must be extractable whenever the process runs (repeatability), and they must respond measurably to changes in process conditions (sensitivity). To test these properties, two controlled deviations from the baseline were introduced, as described in Section 4: a shift in the converter excitation frequency (FS condition) and a reduction in substrate temperature (TS condition).

**Figure 9:**
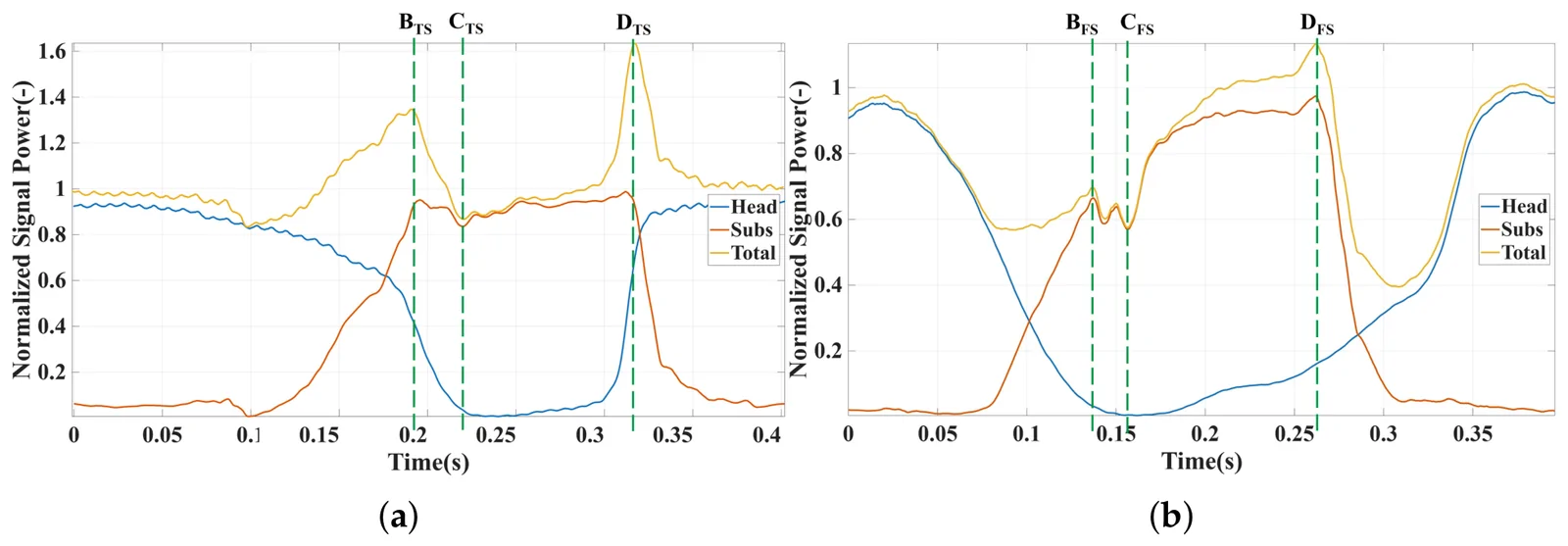
Average normalized power curves of structure–borne AE signals for (**a**) Temperature–Shifted (TS) and (**b**) Frequency–Shifted (FS) conditions.

The average signal power curves for the FS and TS conditions were computed in the same way as for the BC condition in Section 5.1, with the microphone excluded. The resulting curves from five random cycles for each condition are presented in Figure 9. As in the BC scenario, the head and substrate curves follow opposite trends for most of each cycle, interrupted by a distinct interval in which this trade-off no longer holds.

Applying the segmentation criterion of Section 3.3, this interval is resolved into the ISD, where both power curves decrease simultaneously, and the ICD, where both subsequently increase; the corresponding markers are denoted using the same *B*, *C*, and *D* notation, subscripted by the respective process condition. Figure 9, therefore, shows that the trend on which the ISD and ICD are defined persists under altered process conditions, so both features remain extractable when the excitation frequency and substrate temperature are changed. A visual comparison of the average power curves also indicates that the process variations change the length of the windows.

To compare the ISD, ICD, and $T_{IA}$ across print scenarios, the features were extracted from individual deposition cycles and pooled within each condition. Each print attempt contained approximately 8–9 cycles, yielding potential pools of 44, 41, and 42 cycles for the BC, TS, and FS conditions, respectively. Reliable feature extraction was not possible for every cycle, particularly under the FS condition, where the power profiles differed noticeably from those observed under BC and TS, as shown in Figure 9. Features were successfully extracted from 42, 39, and 34 cycles, corresponding to success rates of approximately 95, 95, and 81%. To maintain equal sample sizes for comparison, 34 cycles were therefore retained for each condition. Possible reasons for the altered power–curve behavior under the FS condition are discussed in Section 6.

**Figure 10:**
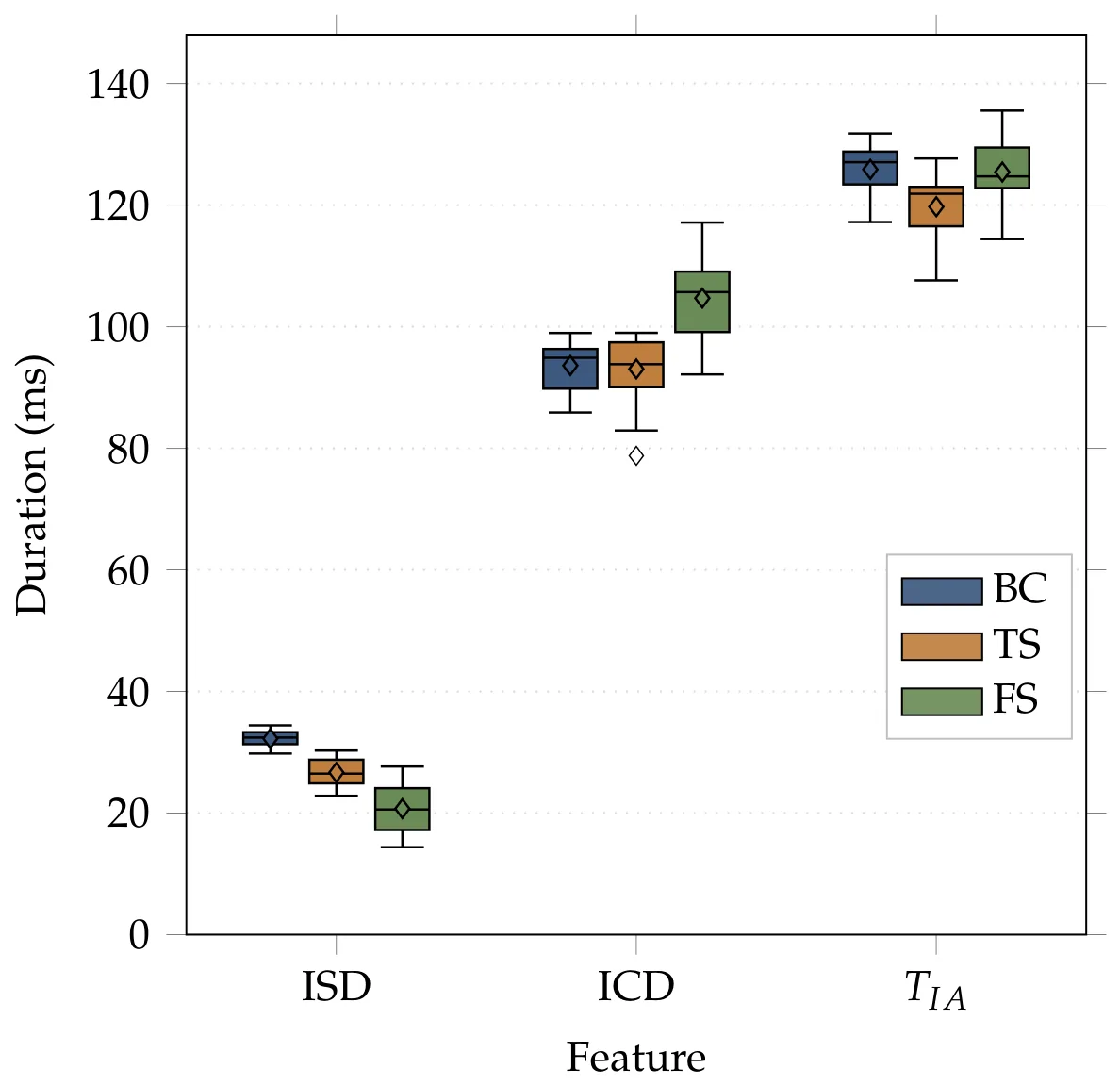
Distribution of the ISD, ICD, and interfacial–activity duration across 34 deposition cycles for each of the three process conditions. Boxes span the interquartile range (IQR), whiskers extend to the most extreme observations within 1.5× IQR, the horizontal line marks the median, and the diamond marks the mean.

The resulting statistics are reported in Table 1, with the corresponding distributions shown in Figure 10. The within–condition coefficient of variation remains low throughout: below 5% for $T_{IA}$ in all three conditions, and between 4 and 9% for the ISD and ICD under BC and TS. The only substantial exception is the ISD under the FS condition, which reaches 19%, consistent with the wider box and whiskers seen in Figure 10. Since these cycles span all five print attempts rather than a single representative deposit, the distributions indicate that the extracted features hold relatively consistent durations across repeated prints when they can be identified.

The same statistics show that the features respond to process variation. The mean ISD decreases monotonically from 32.26 ms at the baseline to 26.68 ms under the reduced substrate temperature and 20.73 ms under the frequency shift, a progression visible as the stepped ISD boxes in Figure 10. The separation between conditions is larger than the scatter within them: the means differ by up to about 11 ms against within–condition standard deviations of 1.4 to 3.9 ms, and the interquartile ranges of the three conditions do not overlap. The ICD behaves differently: it is nearly unchanged by the temperature change, with BC and TS yielding 93.61 and 93.05 ms, but extends to 104.70 ms under the frequency shift. The two features, therefore, move in opposite directions under FS, where a shorter ISD is accompanied by a longer ICD.

Because the five attempts of each condition were printed with a single sensor mounting (Section 4), the BC features were also compared across successive attempts using all 42 successfully segmented cycles to evaluate if the mounting has been violated beyond the visual inspection happening after each print. This data is provided in Table 2, where values are mean ± standard deviation over the *n* successfully segmented cycles of each attempt, for a total of 42 cycles with a single sensor mounting. According to the computed data, the mean of every attempt lies within one standard deviation of the pooled mean, and the differences between the first and last attempts are +0.25, −0.64, and −0.39 ms for the ISD, ICD, and $T_{IA}$, respectively. The ISD coefficient of variation within each attempt remains between 3.4 and 6.2%, with no widening from the first attempt to the last.

**Figure 11:**
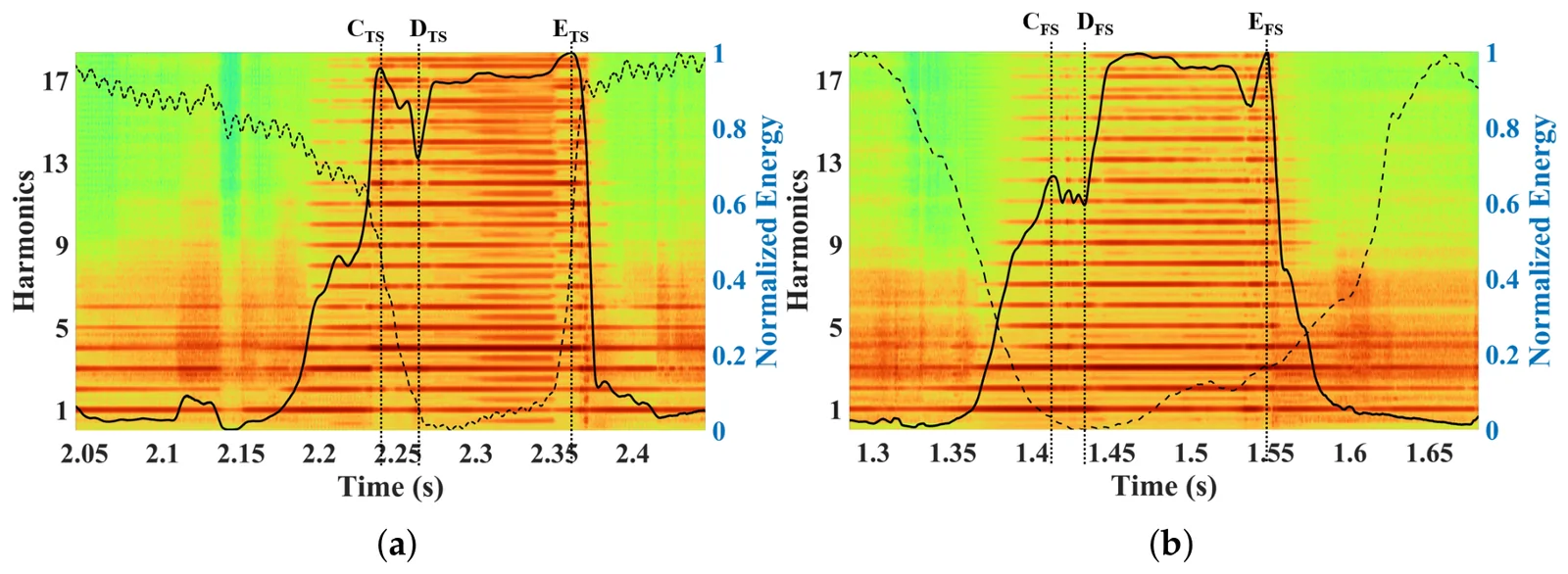
The overlay of normalized head–mounted AE power profile (dashed line) and substrate-mounted AE power profile (solid line) with spectrograms of the substrate–mounted AE signals for modified process conditions: (**a**) Temperature–Shifted (TS) and (**b**) Frequency–Shifted (FS). The marked *B*–*C*–*D* points identify the ISD and ICD intervals that make up the interfacial–activity window.

Beyond the quantitative comparison of ISD and ICD durations, the spectral content of the extracted windows was also examined under the altered conditions. Figure 11 presents representative spectrograms of a single deposition cycle from the substrate–mounted AE sensor for both TS and FS conditions, overlaid with the corresponding normalized head (dashed line) and substrate (solid line) power curves. The marked *B*–*C*–*D* points define the ISD and ICD intervals using the same segmentation criterion.

During the ISD window, higher harmonics appear in both the TS and FS spectrograms, whereas ultra–harmonics appear only in the FS spectrogram and at much lower intensity. The main difference between the two conditions occurs within the ICD window, where the ultra-harmonics have lower magnitudes in the FS spectrogram, whereas in the TS spectrogram, they emerge with a slight delay. In both conditions, the ultra–harmonic content falls within the interval delimited by the power–derived *B* and *D* points.

### 5.4. Similarity Between Airborne and Structure–Borne Signals

Although the microphone data were excluded from the segmentation due to their spatial dependence, the power and spectrogram results showed that the airborne response becomes temporally aligned with the Coupling windows and develops harmonic and ultra-harmonic components when the deposition head is closer to the microphone. The analysis below examines this similarity further in a single baseline cycle.

**Figure 12:**
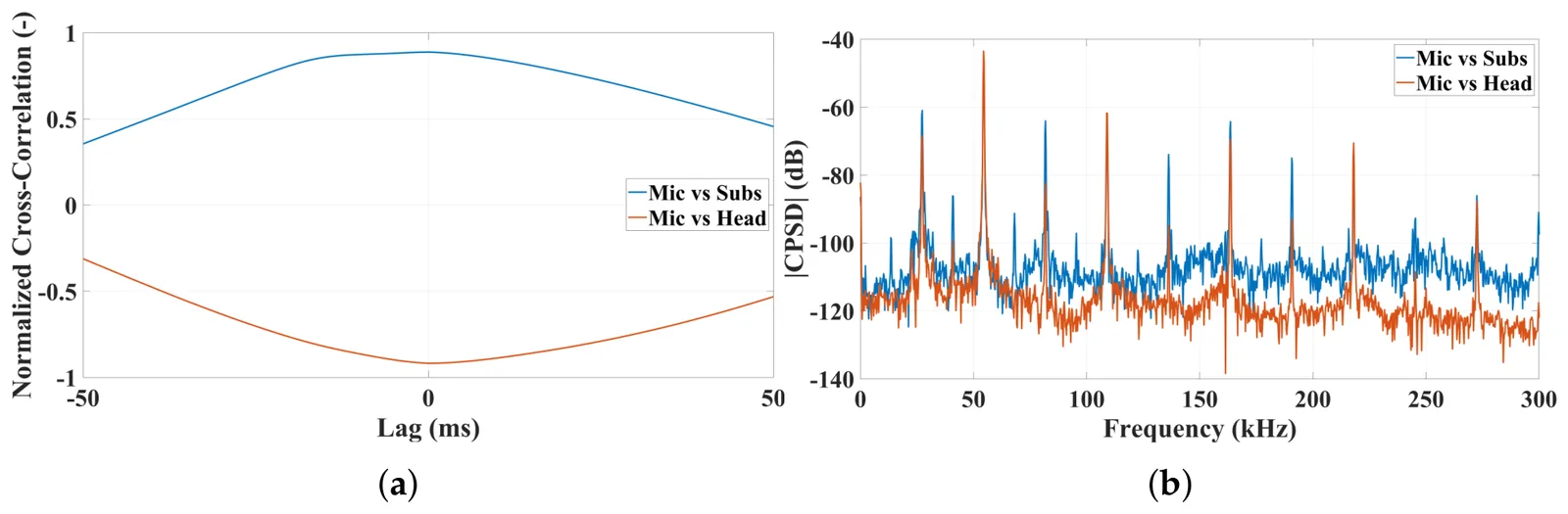
Similarity analysis between the airborne microphone signal and structure–borne AE signals for the selecte deposition cycle: (**a**) normalized cross–correlation of the signal power curves and (**b**) magnitude of the cross–power spectral density, |CPSD|.

A baseline segment was selected in which the microphone was estimated to be closest to the active deposition region, corresponding to one deposition cycle between approximately t=1.78 and 2.14 s. Within this window, the microphone signal was compared with the substrate– and head–mounted AE signals using two complementary measures. Temporal similarity was assessed from the normalized cross–correlation of the signal power curves of the microphone and each AE sensor [37]. For two channels *a* and *b*, the normalized cross–correlation at lag *m* is

(7) $$\rho_{ab}\left[m\right]=\frac{\sum_{n}\left({\tilde{P}}_{a}\left[n\right]-\mu_{a}\right)\left({\tilde{P}}_{b}\left[n+m\right]-\mu_{b}\right)}{\sqrt{\sum_{n}{\left({\tilde{P}}_{a}\left[n\right]-\mu_{a}\right)}^{2}\sum_{n}{\left({\tilde{P}}_{b}\left[n\right]-\mu_{b}\right)}^{2}}}$$

where ${\tilde{P}}_{a}$ and ${\tilde{P}}_{b}$ are the signal power curves of the two sensors, $\mu_{a}$ and $\mu_{b}$ are their time-averages, and $\rho_{ab}\left[m\right]\in \left[-1,1\right]$. This measure tests whether the airborne sensor follows the same temporal evolution as the structure-borne responses; the similarity is summarized by the extrema of $\rho_{ab}\left[m\right]$ and the lag at which it occurs, shown in Figure 12a.

Spectral similarity was assessed from the magnitude of the cross-power spectral density (CPSD) [38]. Dividing each signal into *K* overlapping, tapered segments and denoting by $X_{a}^{\left(k\right)}\left(f\right)$ the discrete Fourier transform of the *k*-th segment of sensor *a*, the CPSD is estimated as

(8) $${\hat{S}}_{ab}\left(f\right)=\frac{1}{K\;U\;f_{s}}\sum_{k=1}^{K}X_{a}^{\left(k\right)}\left(f\right)\;\bar{X_{b}^{\left(k\right)}\left(f\right)},\;U=\sum_{n}g^{2}\left[n\right]$$

where g[n] is the segment window, *U* normalizes for its energy, $f_{s}$ is the sampling frequency, and the overbar denotes complex conjugation. Its magnitude, $|{\hat{S}}_{ab}\left(f\right)|$, identifies the frequencies at which the airborne and structure–borne sensors share correlated power, shown in Figure 12b, and tests whether the shared content includes the harmonics and ultra–harmonics observed within the Coupling windows.

The cross–correlation curves in Figure 12a show that the microphone power is strongly and positively correlated with the substrate response and strongly negatively correlated with the head response. Because the head and substrate power curves are themselves anti–correlated, the microphone cannot be in phase with both, and the sign of the correlation shows that it follows the substrate. The extrema of both curves occur at essentially zero lag, so the airborne and structure–borne responses are temporally aligned to within the resolution of the power–based measure, which is too coarse to resolve the much smaller airborne propagation delay.

The |CPSD| in Figure 12b is shown up to 300 kHz, above which the airborne response becomes weak relative to the structure–borne channels, and the shared content is no longer reliable for this assessment. The microphone spectrum in Figure 6c also confirms that the interpretable components lie primarily below this range. Within this range, the microphone exhibits clear spectral overlap with both AE channels at the integer harmonics of the ultrasonic excitation, with the head leading the substrate by about 4 dB at the fundamental.

The microphone also shares content with the substrate at ultra–harmonic frequencies, and does so far more strongly than with the head. At 27.25, 81.75, 136.25, and 190.75 kHz, the microphone–substrate |CPSD| exceeds the microphone–head value by roughly 7, 18, 21, and 18 dB, respectively, and the same ordering persists across the higher ultra–harmonics.

## 6. Discussion

The results in Section 5 describe the same interval of each deposition cycle from three directions: the signal power of the structure-borne channels, the spectral content of the signals, and the behavior of the proposed features across repeated prints and altered process conditions. This section brings these observations together to explain what the ISD and ICD represent, what they currently offer for monitoring the RAD process, and what remains needed before they can be linked to joint quality. The airborne measurements are discussed afterward.

Outside the interfacial–activity window, the head and substrate powers vary in opposition, which is the redistributive behavior expected from the converter–driven field (Section 3.3). As the head approaches the substrate between *A* and *B*, waves begin to transmit into the substrate while the head retains less of the energy, so one pathway gains what the other loses. The head power never approaches zero, since the converter drives the tool continuously.

Between *B* and *C*, however, both powers fall together. A redistribution between the two pathways cannot produce a simultaneous drop, so this interval indicates that less power reaches both sensors, which is consistent with mechanical loading and dissipation at the contact, as the tool vibration is limited by the substrate. Three observations support the attribution of this interval to interfacial slipping. First, it coincides with the portion of the programmed toolpath in which the tool is commanded into contact with the substrate, so it occurs while the interface is engaged rather than during free tool motion. Second, the direction of the change, a common loss of power on both channels, is what a contact would produce. Third, its duration, 32.26 ms on average across the baseline cycles, is close to the approximately 30 ms of the localized 5 °C temperature rise attributed to the slip stage under comparable conditions [39]. Taken together, these independent observations place a dissipative interfacial process at the point in the deposition cycle where slipping is expected, so the ISD is attributed to interfacial slipping. Although the relative motion at the interface was not measured directly in this work, the agreement between the acoustic, toolpath, and thermal evidence provides a consistent basis for this attribution, which direct observation of the interface can further confirm.

From *C* to *D*, both powers rise together. A change in coupling can attenuate both pathways, but it is unlikely to amplify both, so the common increase indicates an additional acoustic contribution arising at the interface. This agrees with the stages described in Section 2: as slipping subsides, the cleaned surfaces stick, and the joint consolidates through plastic deformation, followed by elastic recovery as the compressive load is released. Plastic deformation is a known source of acoustic emission, so the consolidating joint is likely to act as a secondary source that can raise the total acoustic energy and signal power on both sensors. The behavior after *D* supports this reading. Once the tool detaches from the substrate, the opposing trend between the head and substrate returns rather than a further common rise, indicating that the common increase depends on the interaction at the interface rather than on the converter-driven field alone. The power–based segmentation thus divides the interfacial activity in each cycle into a stage marked by a common loss of power and a stage marked by a common gain, which is the basis of the ISD and ICD.

The spectral results give an independent view of the same interval. The integer harmonics of the excitation are not specific to contact, as they are the only spectral pattern observed during the Retained phases, where no contact is established, identifying the ultrasonic converter as their source. Accordingly, the harmonics shift between the head and substrate spectrograms in the same way as the signal power, following the redistribution of the converter-driven field. The ultra–harmonics behave differently. They appear only within the Coupling windows, are strongest on the substrate, emerge mainly toward the end of the ISD, and persist throughout the ICD, so their onset lies close to the transition from a common loss to a common gain of energy identified in the time domain. Harmonic and secondary frequency content has also been reported in ultrasonic metal welding [19,21,27], which indicates that such signatures are common to ultrasound-based joining rather than specific to RAD.

For monitoring, the two analyses complement each other. The proposed signal power features determine where the interfacial activity lies within each cycle, and the time–frequency analysis can focus only on that interval. Because much of the harmonic variation in the full spectrograms follows the tool motion and the redistribution of the converter–driven field, confining the time–frequency analysis to the *B*–*D* interval separates the interface–related content from these variations and reduces the computational cost. Under the TS and FS conditions, the ultra–harmonics differ in magnitude and timing, yet they still fall within the introduced intervals, so the proposed time–based features continue to isolate the spectrally informative part of the cycle when the process changes.

The repeatability results indicate that the features can be consistently obtained. Pooled across all five print attempts, the coefficient of variation of $T_{IA}$ remains below 5% in all three conditions, and that of the ISD and ICD remains between 4 and 9% under BC and TS. Since the contact sensors were taped to surfaces near 100 °C, their coupling could in principle change over successive attempts, yet the baseline features show no systematic drift from the first attempt to the last. This is expected given how the features are defined: the ISD and ICD are transition timings rather than amplitude measures, and each channel is normalized within the cycle, so a change in coupling that alters the level of a power curve does not displace its extrema in time. The features are also sensitive to the process in a way that separates the two deviations. Reducing the substrate temperature shortens the ISD while leaving the ICD essentially unchanged, whereas shifting the excitation frequency shortens the ISD and lengthens the ICD. As a result, $T_{IA}$ is nearly identical under BC and FS (125.87 and 125.44 ms), since the shorter ISD is offset by the longer ICD. It is therefore the division of the interfacial activity into two stages, rather than its total duration, that reveals the frequency shift.

The extraction success rate also reflects the process state. Its drop from about 95% under BC and TS to 81% under FS indicates that, in some cycles, the frequency shift alters the power curves enough that the transitions defining *B*, *C*, and *D* can no longer be distinguished. Forcing the converter to excite at 54 kHz instead of 54.5 kHz moves it away from its baseline operating point, and detuning of the system resonance is a likely consequence. This can alter the vibration amplitude and phase, the mechanical coupling at the interface, and the acoustic transfer between the head and substrate, all of which the measured response depends on (Section 2). These changes can reshape the head and substrate power profiles and reduce the contrast between the common–mode decrease and increase within the Coupling window. Accordingly, the segmentation criterion applies to cycles in which the characteristic cycle structure is preserved, namely an opposing head–substrate power trend outside the interfacial–activity window and a resolvable common–mode decrease followed by a common–mode increase within it. Cycles that do not show this structure are not assigned ISD or ICD values, so the extractability of the features is assessed separately from their repeatability once extracted, and a drop in extractability can itself indicate that the process has departed from the baseline condition.

Taken together, the energy–based analysis, spectral investigations, and statistical results support the introduction of ISD and ICD as time–domain features that locate and time interfacial activity in every deposition cycle using fixed sensors, independent of the tool position. Although extracted after each print in the present study, the features are suited to in–process monitoring, as they become available once the Coupling window closes within a deposition cycle of approximately 360 ms, and their extraction is computationally light and automated using a moving average of the squared waveform and the locations of three extrema. Considering the chain that connects an acoustic feature to a process condition, then to manufacturing quality, and finally to a corrective action, the present work establishes the first two links: the features segment each cycle into distinct stages, and they respond in distinguishable ways to controlled changes in excitation frequency and substrate temperature. Building on these links, the next two steps can be achieved. Relating the ISD and ICD to measured quality metrics, such as bond strength or cross–sectional bonded area, can be done through destructive or metallographic tests, and it is considered as future work. In addition, since the examined process deviations produce distinct feature signatures, the features could support closed–loop control, in which a departure from the baseline signature is detected, and the process is returned to its baseline condition. The interpretation of the two intervals as slipping and consolidation stages can also be further verified through direct observation of the interface, for example, through high–speed imaging, which has been used for this purpose in ultrasonic metal welding [28,29,30].

The airborne results should be read against these features. In the baseline cycle in which the microphone was favorably positioned, its power followed the substrate in time and varied in opposition to the head, and its cross–spectrum with the substrate exceeded that with the head at the ultra–harmonic frequencies by roughly 7 to 21 dB. At the integer harmonics, the microphone shared content with both AE channels, with the head leading, as expected, because the airborne channel records the radiation from the converter-driven vibration that originates at the head. The preferential sharing at the ultra-harmonics indicates that the airborne response is not only the radiated drive tone but also carries the substrate’s acoustic signature within the Coupling windows. This supports the feasibility of detecting interfacial activity with a non–contact sensor.

While the findings indicate that deliberate microphone placement can mitigate the limitations associated with source–to–sensor distance, several challenges remain. Microphones generally have a narrower bandwidth compared to structure–borne acoustic sensors, causing some high–frequency components to fall outside the calibrated measurement range. In addition, airborne measurements are susceptible to ambient noise in the manufacturing environment, whereas structure–borne sensors are comparatively isolated from it. Airborne sensing is therefore regarded here as a complementary measurement rather than a complete replacement for substrate–mounted sensing. Nevertheless, it may play an important role in large–scale or geometrically complex builds, where substrate–mounted sensing can be compromised by propagation attenuation, varying source–to–sensor distance, or limited access to the build surface.

## 7. Conclusions

This study introduced an energy–based acoustic framework for detecting and timing the interfacial activity events during the RAD process, an ultrasound-based additive manufacturing technique. The method treats the recorded signal power of each channel as a relative indicator of acoustic propagation and redistribution among the deposition head, the substrate, and the surrounding air, thereby enabling interfacial activities to be tracked with fixed sensors regardless of the tool position.

The major findings of this work can be summarized as follows:

1. The multi–sensor configuration locates, within each deposition cycle, the interval during which the interface is acoustically active. Although the individual pathways cannot be measured in absolute terms, the onset and progression of interfacial activity are inferred from the correlated, common–mode variation in the head and substrate signal powers, thereby acoustically identifying the interfacial activities.
2. Two time–domain features segment this window and mark the successive stages of the bonding event: the ISD, associated with the slip stage, and the ICD, encompassing the subsequent stick–slip, deformation, and elastic recovery. Across the investigated conditions, the ISD ranged from approximately 21 to 32 ms and the ICD from 93 to 105 ms, giving a combined interfacial-activity duration $T_{IA}$ of approximately 120 to 126 ms. The measured ISD is consistent with an independently reported slip–stage thermal response, supporting the interpretation of the segment as a real interfacial event.
3. The spectral and time–frequency analyses revealed harmonics and ultra–harmonics that characterize the nonlinear interfacial dynamics. High–bandwidth AE sensing, rather than conventional vibration measurement, captured a substantially broader frequency range, up to 18 harmonic orders, which is necessary to resolve the higher–order components and ultra–harmonic transitions that flag interfacial activity.
4. Confining the time–frequency analysis to the signal–derived ISD and ICD windows isolates the bonding–related spectral behavior from the harmonic variations produced by tool motion, improving the specificity of the spectral features while reducing computational cost.
5. Across controlled changes in excitation frequency and substrate temperature, the ISD and ICD remained extractable using the same criterion and responded in distinguishable ways, indicating both repeatability and sensitivity. These properties support the method as a physically interpretable, fixed–sensor basis for detecting and characterizing interfacial activities in moving–tool ultrasound–based processes, with potential transferability to other ultrasound–assisted joining systems.
6. A preliminary comparison of the airborne microphone with the structure–borne channels, during a favorably aligned baseline cycle, showed that the microphone follows the substrate response in time and shares its cross–spectral content at the ultra–harmonic frequencies associated with interfacial activity, more so than with the head. Although limited to a single condition and not a replacement for the structure–borne sensors, this indicates that a suitably positioned microphone can retain part of the interfacial activities signature.

## Figures and Tables

**Table 1:** Mean ± standard deviation of the power–derived features across 34 deposition cycles spanning the five print attempts. Values in parentheses are the coefficient of variation (CV).

Feature (ms)	Process Condition		
	*BC*	*TS*	*FS*
ISD	32.26±1.43 (4.4%)	26.68±2.30 (8.6%)	20.73±3.94 (19.0%)
ICD	93.61±3.80 (4.1%)	93.05±5.09 (5.5%)	104.70±7.01 (6.7%)
$T_{IA}$	125.87±3.67 (2.9%)	119.73±4.96 (4.1%)	125.44±5.36 (4.3%)

**Table 2:** Consistency of the extracted features across successive print attempts under the baseline condition.

Print Attempt	*n*	ISD (ms)	ICD (ms)	$T_{\mathrm{IA}}$ (ms)
1	9	31.74±1.31	95.86±3.83	127.60±3.72
2	8	32.22±2.00	93.00±4.60	125.21±4.25
3	9	30.69±1.56	95.54±1.16	126.23±2.19
4	8	32.10±1.25	92.54±4.70	124.64±3.83
5	8	31.99±1.08	95.22±3.23	127.21±3.56
All attempts	42	31.72±1.51	94.49±3.77	126.21±3.56
First vs. last attempt	–	+0.25	−0.64	−0.39

## Data Availability

The data presented in this study are available on request from the corresponding author.

## Abbreviations

The following abbreviations are used in this manuscript:

AE	Acoustic Emission
BC	Baseline Condition
CPSD	Cross-Power Spectral Density
CV	Coefficient of Variation
DED	Directed Energy Deposition
FDM	Fused Deposition Modeling
FFT	Fast Fourier Transform
FS	Frequency-Shifted condition
ICD	Interfacial Consolidation Duration
IQR	Interquartile Range
ISD	Interfacial Slipping Duration
LPBF	Laser Powder Bed Fusion
NDE	Nondestructive Evaluation
RAD	Resonant-Assisted Deposition
STFT	Short-Time Fourier Transform
TS	Temperature-Shifted condition
USMW	Ultrasonic Metal Welding
WAAM	Wire Arc Additive Manufacturing
